# Notes and News

**Published:** 1903-11-14

**Authors:** 


### NOTES AND NEWS.

The Princess Louise has given her patronage to the bazaar in aid of the Victoria Hospital for Children, which is to be held in the Albert Hall. Princess Henry of Battenberg will open a bazaar on the 18th inst. in aid of the Glasgow Samaritan Hospital.

At the inquest held on the body of the late Miss Sophia Hickman, M.D., Dr. Thomas Stevenson stated that he found very definite traces of morphine in the viscera.

The members of the Vienna Medical Chamber, which is entrusted with the professional control of doctors in the city, resigned *en masse* in consequence of accusations brought against medical men in Vienna by members of the Landtag, that they had carried on vivisection improperly.

A quantity of pure radium has been generously placed at the disposal of a member of the staff of the Cancer Hospital, Fulham Road, S.W., and the staff are making arrangements for an extensive trial of this new method of treatment to be undertaken. With the number of inoperable cases at their disposal the staff will be able to give this new treatment a fair and exhaustive trial.

Lord Dartmouth is appealing very urgently for more hospital accommodation in South London. He is President of the Bolingbroke Hospital at Wandsworth, a little hospital with only thirty beds, which has been striving valiantly for many years to help the large poor population in its vicinity. There can be no doubt of the useful and self-sacrificing work done there, and in which Canon Erskine Clarke has taken so large a part. The district is extremely poor, but it recognises and appreciates the efforts of the hospital, and £2,600 out of the local Coronation Commemoration Fund is to be devoted to extending the accommodation. Lord Dartmouth points out that if those who are well to do outside the district would supplement the local efforts to the extent of £5,000 twenty beds could be added to those now existing. They are sorely needed.

A pretty drawing of the proposed buildings for the King's Sanatorium was published in the *Graphic* for November 7th.

We congratulate Mr. J. G. Craggs upon the honour of knighthood just bestowed upon him by the King. Mr. Craggs, who is a Companion of the Victorian Order, has proved himself a zealous honorary secretary of the King's Hospital Fund from its commencement, and he has shown his genuine interest in the advancement of medical science by the generous support which he has afforded to the work of the London School of Tropical Medicine, in connection with which he has maintained a Craggs Scholarship for some years. He recently took up the cudgels of voluntary *versus* State-supported hospitals in an able letter to the *Times*, which he subsequently elaborated and republished in pamphlet form.

The Craggs research prize, for the best piece of original work done during the current year by present or past students of the school, has been awarded to Dr. Aldo Castellani for his researches into the etiology of sleeping sickness. It will be remembered that Dr. Castellani isolated a peculiar streptococcus in sleeping sickness, and was the first to point out the association of trypanosomes with this disease, having demonstrated their frequent presence in the cerebro-spinal fluid as well as in the blood. Dr. Travers, who also competed for the prize, has been awarded honourable mention for his paper on beri-beri.

The death of the Polar bear which has inhabited the Zoological Gardens since 1895, of aneurysm of the aorta, is of considerable interest from a pathological point of view. The text-books usually mention syphilis, alcoholism, and physical overstrain as the chief causes of aneurysm, apart from local injury. In the case of the Polar bear the first two may be safely excluded, and although we are not acquainted with his habits previous to his entry to the Zoo in 1895, his life since that date seems to have been peaceful enough to allow the exclusion of physical overstrain. Therefore some other cause remains to be revealed, and this, if applicable to the human, may give rise to issues of the highest importance.

It appears from a letter from the London Hospital to the School Board, that an immense and increasing number of children have presented themselves recently for the purpose of having their eyesight tested. The letter concludes by stating that the hospital cannot undertake this work in future, as they regard it as a matter for the Board's medical officers. The Board replied that they did not directly send children to any one hospital, but that nevertheless the hospitals must count upon a large increase in eye cases attending the out-patients' departments owing to the increased medical supervision exercised by the School Board, and the hospitals would either have to increase their staff or the rates would have to undertake the responsibility. The London Hospital again replied that, as the East End hospital, the strain upon its resources was too great, and that it could not do the work. The matter is still under consideration.

